# Associations of anxiety and depression with restless leg syndrome: a systematic review and meta-analysis

**DOI:** 10.3389/fneur.2024.1366839

**Published:** 2024-03-18

**Authors:** Tianyang An, Haiyang Sun, Lu Yuan, Xiuling Wu, Baoquan Lu

**Affiliations:** ^1^College of Clinical Medicine, North China University of Science and Technology, Tangshan, Hebei, China; ^2^Cangzhou Center for Disease Control and Prevention, Cangzhou, Hebei, China; ^3^College of Psychology and Mental Health of North China University of Science and Technology, Tangshan, Hebei, China; ^4^Department of Neurology, Tangshan Gongren Hospital, Tangshan, Hebei, China

**Keywords:** depression, anxiety, restless legs syndrome, meta-analysis, mental health

## Abstract

**Background:**

The levels of anxiety and depression among patients with restless leg syndrome (RLS) are controversial. The aim of this systematic review and meta-analysis was to compare the levels of depression and anxiety among individuals with RLS with those of healthy controls.

**Methods:**

We conducted an extensive electronic search of the PubMed, Web of Science, EMBASE, and Cochrane Library databases from their inception dates to 20 June 2023. Studies presenting data on depression and anxiety in individuals with RLS were included, and a comprehensive meta-analysis was performed.

**Results:**

Twenty-one studies matched the inclusion criteria. Significantly more depressive symptoms were present in the individuals with RLS than in those without RLS, as measured by the Beck Depression Inventory [mean difference (MD) = 6.58, 95% confidence interval (CI) = 5.54–7.62, *p* < 0.01; heterogeneity I^2^ = 0%, *p* = 0.99]. Similarly, the results from the Beck Anxiety Inventory indicated that there were significantly more pronounced anxiety symptoms in the individuals with RLS than in those without RLS (MD = 9.30, 95%CI = 7.65–10.94, *p* < 0.01; heterogeneity I^2^ = 0%, *p* = 0.92). The other anxiety and depression scales also yielded statistically significant results. Significant heterogeneity was observed in the Hamilton Depression Rating Scale and Hamilton Anxiety Rating Scale, with the primary contributing factor probably being the scoring criteria of the scales.

**Conclusion:**

This meta-analysis found that the levels of depression and anxiety symptoms were significantly higher in individuals with RLS than in their healthy counterparts.

**Systematic review registration:**https://www.crd.york.ac.uk/prospero/display_record.php?ID=CRD42023410364, (identifier CRD42023410364).

## 1 Introduction

Restless Legs Syndrome (RLS) is the most common sleep-related sensorimotor disorder characterized by an irresistible urge to move the legs, with typical worsening of symptoms at night during periods of immobility. In the general population, the prevalence is approximately 5–15%, with female predominance (1–3). The prevalence also increases with age in European and North American countries, but not in Asian countries (4). The disease is divided into two types: primary RLS, also known as idiopathic RLS, and secondary RLS, which may be linked to various factors, such as pregnancy, iron deficiency, end-stage renal disease, or neuropathy. Both types share the same symptoms and diagnostic criteria (5). The pathogenesis of primary RLS has not been fully established; however, genetic factors, impaired dopaminergic neuron function, and brain iron deficiency are recognized as contributors to its development (6).

Symptoms of anxiety and depression are consistently associated with RLS. Previous studies have reported that major depressive disorders, anxiety, and abnormal personality traits, such as neuroticism, are common in RLS (7, 8). Compared with their nonaffected counterparts, individuals with RLS are more likely to suffer from anxiety, depression, chronic pain, and cognitive difficulties. Individuals with RLS exhibit cognitive deficits in the domains of short-term attention, verbal fluency, visuospatial functions, and executive function (9–11). Furthermore, RLS, with its high prevalence, significantly affects emotional well-being and elevates the risk of cardiovascular diseases, ultimately affecting overall quality of life (12). Epidemiological studies have reported a two-to fourfold higher risk of depressive disorders in individuals with RLS than in healthy controls (13, 14). Consequently, RLS has become a nervous system disorder that is closely related to sleep, mood, cognition, and productivity in individuals’ daily lives. Furthermore, as the understanding of RLS continues to expand, comprehensive evidence regarding mental health outcomes will have an impact on mental health care (15, 16).

Although a previous narrative review provided an overview of RLS diagnosis and reported epidemiological evidence for an association between RLS and mood disorders (17), the association between RLS and depression/anxiety has been less well studied. Therefore, our study aim was to conduct a systematic review and meta-analysis to compare the levels of depression and anxiety between individuals with RLS and healthy controls.

## 2 Methods

### 2.1 Registration

This meta-analysis was registered in the International Prospective Register of Systematic Reviews (PROSPERO CRD42023410364) and was conducted in accordance with the Cochrane guidelines.

### 2.2 Study search

We searched the PubMed, Web of Science, EMBASE and Cochrane Library, from inception date through 20 April 2023, and further updated the search on 20 June 2023. We utilized the Medical Subject Headings (MeSH) term “restless legs syndrome” combined with the following free text terms: “anxiety” and “depression.” No restrictions were placed on geographic location, language, or availability of the full text. To extend the scope of the search, we also meticulously reviewed the references of relevant review and meta-analysis studies to find additional articles of interest. The references were managed using Endnote software.

### 2.3 Study selection (inclusion and exclusion criteria)

Search results were imported into Endnote, where duplicates were removed automatically. Two authors (Tianyang An and Haiyang Sun) conducted a two-stage review process. Titles and abstracts were evaluated at the first stage and peer-reviewed full-length studies that met the following predefined criteria were considered for inclusion at the second stage. These criteria included: (1) observational study (cross-sectional, case–control, cohort); (2) the study’s population should have been stratified into at least two groups: cases (with RLS) and controls (without RLS); (3) the exposure of interest was depression or anxiety symptoms, or both; and (4) separately reported mean values (M) with their standard deviations (SD) for depression or anxiety symptoms, or both, using a validated quantitative scale for both cases (with RLS) and controls (without RLS). We excluded studies as follows: (1) RLS studies with multiple simultaneous groups; (2) studies that did not have a control group; and (3) conference abstracts, meta-analysis and reviews, animal studies, case series, case reports, or letters; and (4) studies that did not compare across groups or failed to address the primary research question.

### 2.4 Data extraction

Two authors (Tianyang An and Haiyang Sun) independently extracted the following data to a customized data extraction form eligible studies: publication year, first author, country of origin, study type, mean age pf participants, percentage of female participants, sample size, data resources, and depression/anxiety scores. In cases where certain data were not readily available in the included studies, we contacted the corresponding author of the original study to request unavailable data.

### 2.5 Study quality assessment

Using the Newcastle–Ottawa Scale, two authors (Tianyang An and Haiyang Sun) independently evaluated the quality of each eligible study, considering case–control, cross-sectional, and cohort study designs, into high quality (7–9), moderate quality (4–6), or low quality (0–3) (18). Any discrepancies were resolved through an objective discussion with the third author (Lu Yuan).

### 2.6 Statistical analyses

Review Manager software 5.4.1was used to analyze the data. Mean (M), SD, and mean difference (MD) with corresponding 95% confidence interval (CI) were used to compare depression and anxiety symptoms between individuals with RLS and those without RLS. The pooled point estimates of the MDs of the average scores of depression and anxiety, with 95%CIs, were calculated using forest plots, where the size of the square represents the weight of each study and the horizontal lines represent the 95%CI. We considered a statistically significant difference to be present when the 95%CI did not encompass zero. The heterogeneity of the studies was assessed graphically with forest plots and statistically via a chi-square-based Q statistic and I^2^ value. *p* < 0.05 was considered statistically significant (19). To investigate and mitigate sources of heterogeneity or inconsistency, we conducted subgroup analyses and several sensitivity analyses. All sensitivity analyses were carried out using STATA v.12.0 (Stata, College Station, TX, United States). In addition, funnel plots and Egger’s test were used to explore the potential publication bias in this meta-analysis (20, 21).

## 3 Results

### 3.1 Search results

**Figure 1:**
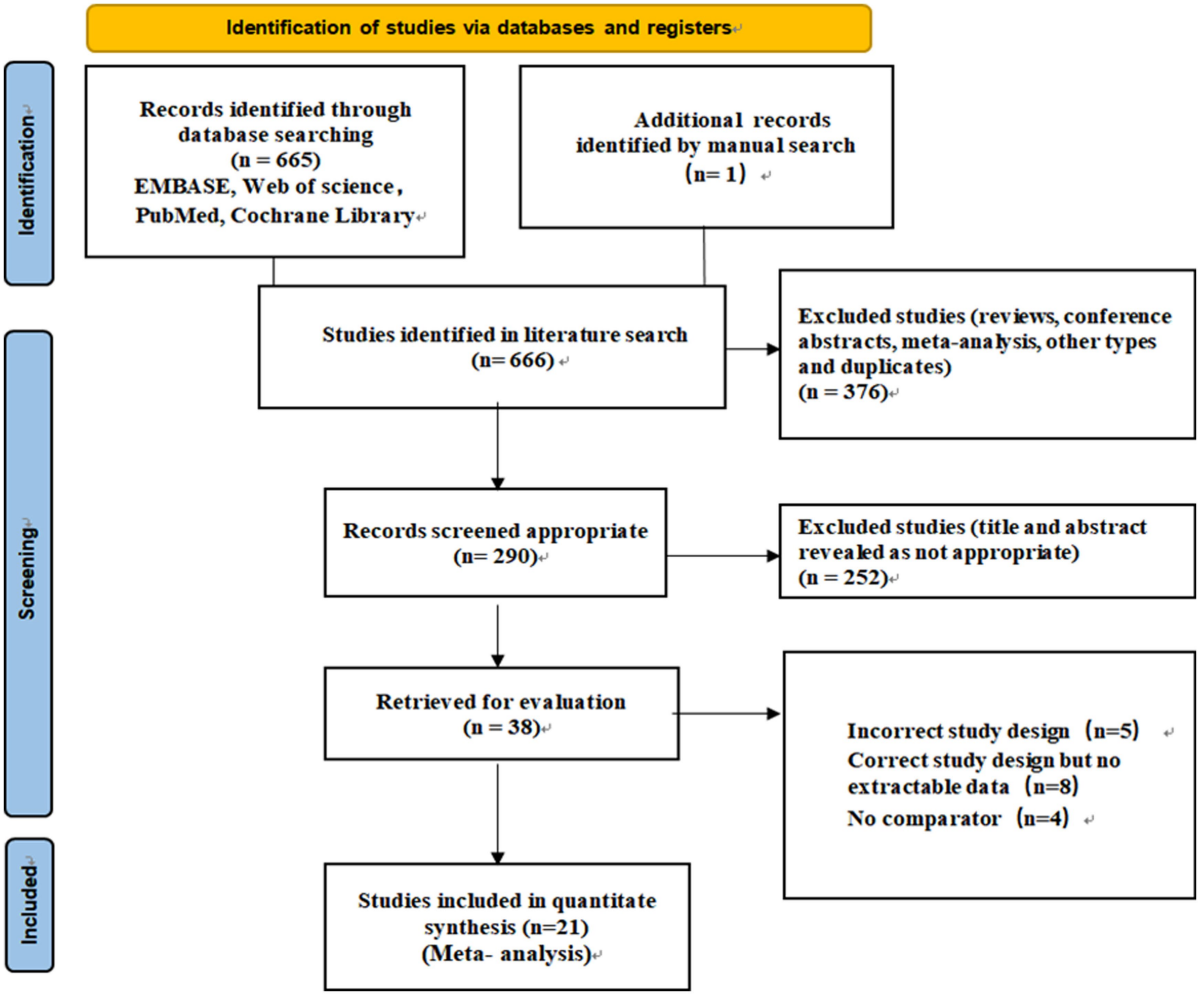
PRISMA flow diagram of identification, screening, and selection process for the meta-analysis on depression, anxiety, and Restless Legs Syndrome.

As shown in Figure 1, our search strategy resulted in a total of 666 original studies. After the removal of reviews, conference abstracts, meta-analysis, other types of records, and duplicates, we identified 290 articles that appeared relevant to the study question. Following the subsequent title and abstract screening, 38 articles remained for full-text review. Articles that were excluded during full-text review were: study designs that did not match inclusion criteria (*n* = 5); experimental designs meet criteria but without extractable data (*n* = 8); and lack of a control group (*n* = 4). In total, we included 21 studies in the meta-analysis.

### 3.2 Study characteristics

A summary of the 21 included study characteristics was shown in Table 1. The studies included a total of 1,626 individuals with RLS and 8,824 individuals without RLS. Table 2 summarized the study qualities where all studies received a rating of high quality.

**Table 1:** Basic characteristics of included studies in meta-analysis.

Publish year	First Author	Country	Study type	Age	Study group	Sample size	Percentage of female	Mean age	Depression	Anxiety
									BDI	TEMPS-A
2021	Sehnaz Basaran	Turkey	Cross-sectional	NA	Rls	74	77%	47.5 ± 11.7	13.6 ± 6.9	6.3 ± 5.2
					Control	90	80%	43.7 ± 9.2	7.1 ± 4.1	3.5 ± 2.6
									TEMPS-A	HAMA
					Rls				6.6 ± 2.9	23.9 ± 10.8
					Control				4.9 ± 2.3	4.9 ± 4.0
									HAMD	HAMA
2004	S Sevim	Turkey	Cross-sectional	>17	Rls	103	62%	NA	9.27 ± 5.03	8.03 ± 6.02
					Control	103	NA	43.1 ± 15.22	5.88 ± 4.99	5.91 ± 6.68
									BDI	BAI
2018	Onur Yilmaz	Turkey	Cross-sectional	≥18	Rls	87	79.3%	46.8 ± 9.3	13.6 ± 7.8	17.4 ± 11.4
					Control	88	76.1%	44.4 ± 10.89	7.4 ± 5.2	9.0 ± 7.1
									Goldberg	Goldberg
2017	M T Barroso-Perez	Spain	Cross-sectional	≥18	Rls	102	79.4%	49.86 ± 15.07	3.1 ± 2.8	4.2 ± 3.5
					Control	138	76.8%	51.39 ± 14.99	1.3 ± 2.0	2.2 ± 2.6
									BDI	BAI
2019	Senay Aydin	Turkey	Cross-sectional	>24	Rls	45	86.7%	38.55 ± 8.438	14.31 ± 8.28	18.29 ± 9.35
					Control	20	65%	39.65 ± 8.857	7.40 ± 10.01	7.70 ± 8.45
									SDS	SAS
2013	J. Steinig	Germany	cross-sectional	NA	Rls	30	70%	57.6 ± 12.6	39.9 ± 7.4	37.4 ± 4.9
					Control	30	NA	NA	28.8 ± 8.6	26.0 ± 0.4.3
									HADS	HADS
2023	Hanife Kocakaya	Turkey	Cross-sectional	NA	Rls	52	73%	34.00 ± 8.27	7.82 ± 4.23	10.07 ± 4.29
					Control	57	58%	31.70 ± 9.12	4.78 ± 3.44	6.28 ± 3.98
									BDI	BAI
2022	Zahide Mail Gurkan	Turkey	Cross-sectional	NA	Rls	80	78.8%	45.41 ± 8.24	14.01 ± 7.76	17.48 ± 11.89
					Control	50	74%	43.12 ± 10.35	6.46 ± 5.96	6.98 ± 7.69
									HAMD	HAMA
2018	Gen Li	China	Cross-sectional	NA	Rls	40	67.5%	57.70 ± 13.93	1.18 ± 1.20	1.72 ± 1.36
					Control	40	67.5%	57.70 ± 13.93	1.10 ± 1.26	1.38 ± 0.98
									BDI	
2019	Jung-Ick Byun	Korea	Cross-sectional	NA	Rls	20	60%	59.5 ± 13.0	16.2 ± 8.9	
					Control	20	50%	57.9 ± 6.7	9.8 ± 5.4	
									BDI	BAI
2016	Nesrin Helvacı Yılmaz	Turkey	Cross-sectional	18~65	Rls	30	66.7%	44.73 ± 11.23	14.03 ± 8.22	15.97 ± 10.46
					Control	30	56.7%	46.00 ± 12.23	8.13 ± 7.95	57 ± 6.56
									SDS	SAS
2014	Marta Fernández-Matarrubia	Spain	Case–control	18~65	Rls	47	57%	51.0 ± 9.6	45.3 ± 13.0	42.7 ± 9.2
					Control	47	57%	51.3 ± 9.6	38.7 ± 9.8	36.8 ± 7.3
									SDS	SAS
2002	Michael Saletu	Austria	Cross-sectional	31~82	Rls	33	54.5%	59.0 ± 11.5	39.9 ± 8.5	36.8 ± 8.4
					Control	33	NA	57.0 ± 11.6	29.6 ± 4.6	26.9 ± 3.8
									HADS	HADS
2016	Jianhua Chen	China	Cross-sectional	≥18	Rls	42	61.9%	54.4 ± 13.3	4.1 ± 3.1	6.6 ± 3.5
					Control	42	NA	NA	0.7 ± 1.3	1.4 ± 1.7
									PHQ-9	BAI
2020	Hee-Jin Im	Korea	Case–control	>19	Rls	18	66.7%	47.3 ± 12.1	7.2 ± 7.2	11.4 ± 12.7
					Control	15	60%	38.8 ± 12.9	2.9 ± 3.2	3.3 ± 4.1
									SCL-90R	SCL-90R
2014	Ellen Trautmann	Germany	(Manual search) cross-sectional	18~85	Rls	47	70.2%	57.5 ± 12.21	12.3 ± 10.1	7.5 ± 6.7
					Control	37	56.8%	55.6 ±11.58	3.4 ± 3.6	2.1 ± 3.06
									QD2A	Goldberg
2010	Sébatien Celle	France	Cohort study	NA	Rls	494	29.3%	68.6 ± 0.8	3.7 ± 3.3	4.6 ± 2.8
					Control	173	70.7%	68.5 ± 0.9	2.1 ± 2.3	2.8 ± 2.7
									CES-D	(Anxiety or depression) EQ-5D
2009	Seong-Jin Cho	Korea	Cross-sectional	18~64	Rls	72	75%	49.5 ± 12.6	10.8 ± 9.4	1.32 ± 0.53
					Control	6,347	60.2%	41.0 ± 12.0	6.4 ± 7.7	1.13 ± 0.35
										STAI
2014	Stefano Zanigni	Italy	Cross-sectional	≥18	Rls	159	60.4%	54.4 ± 13.3		45.8 ± 0.4
					Control	1,408	56.5%	45.5 ± 16.1		45.7 ± 0.1
									GDS	
2014	Hochang Benjamin Lee	America	Cross-sectional	34~98	Rls	23	91.3%	69.6 ± 12.5	10.50 ± 8.07	
					Control	37	75.7%	68.1 ± 8.9	5.19 ± 4.98	
									BDI	BAI
2014	Esra Yancar Demir	Turkey	Cross-sectional	≥18	Rls	28	89.3%	NA	16.8 ± 12.14	19.7 ± 12.20
					Control	19	64.3%	NA	10.6 ± 6.71	11.3 ± 7.60

**Table 2:** Assessment of study quality with Newcastle-Ottawa Scale.

Cross-sectional and case–control studies^1^	Selection				Comparability	Exposure			Total
	S1	S2	S3	S4	C	E1	E2	E3	
Gen Li et al. (9)	*	*	*	*	*	*	*	*	8
Sehnaz Basaran et al. (22)	*	*	*	*	* *	*	*	*	9
S Sevim et al. (23)	*	*	*	*	*	*	*	*	8
Onur Yilmaz et al. (24)	*	*	*	*	* *	*	*	*	9
M T Barroso-Perez et al. (25)	*	*		*	*	*	*	*	7
Senay Aydin et al. (26)	*	*	*	*	*	*	*	*	8
J Steinig et al. (27)	*	*		*	*	*	*	*	7
Hanife Kocakaya et al. (28)	*	*		*	* *	*	*	*	8
Zahide Mail Gurkan et al. (29)	*	*	*		*	*	*	*	7
Jung-Ick Byun et al. (30)	*	*	*	*	*	*	*	*	8
Nesrin Helvaci Yilmaz et al. (31)	*	*	*	*	*	*	*	*	8
Marta Fernández-Matarrubia et al. (32)	*	*		*	*	*	*	*	7
Michael Saletu et al. (33)	*	*	*		*	*	*	*	7
Jianhua Chen et al. (34)	*	*		*	*	*	*	*	7
Hee-Jin Im et al. (35)	*	*		*	*	*	*	*	7
Ellen Trautmann et al. (36)	*	*	*	*	*	*	*	*	8
Seong-Jin Cho et al. (37)	*	*	*	*		*	*	*	7
Stefano Zanigni et al. (38)	*	*	*	*	*	*	*	*	8
Hochang Benjamin Lee et al. (39)	*	*	*	*	*	*	*	*	8
Esra Yancar Demir et al. (40)	*	*	*	*	* *	*	*	*	9
**Cohort studies** ^**2**^	**Selection**				**Comparability**	**Exposure**			**Total**
	**S1**	**S2**	**S3**	**S4**	**C**	**E1**	**E2**	**E3**	
Sébatien Celle et al. (41)	*	*	*	*	*	*	*	*	8

Quality evaluation result. ^1^Newcastle-Ottawa Scale for cross-sectional and case–control studies: Selection contains four criteria: S1, is the case definition adequate? S2, representativeness of the cases; S3, selection of controls; S4, definition of controls. Comparability (C) of cases and controls on the basis of the design or analysis. Exposure contains three criteria: E1, ascertainment of exposure; E2, same method of ascertainment for cases and controls; E3, non-response rate. ^2^Newcastle-Ottawa Scale for cohort studies: Selection contains four criteria: S1, representativeness of the exposed cohort; S2, selection of the non-exposed cohort; S3, ascertainment of exposure; S4, demonstration that outcome of interest was not present at start of study. Comparability (C) of cohorts on the basis of the design or analysis. Outcome contains three criteria: O1, assessment of outcome; O2, was follow-up long enough for outcomes to occur? O3, adequacy of follow up of cohorts.

Of the included studies, eight were conducted in Turkey (22–24, 26, 28, 29, 31, 40), three in Korea (30, 35, 38), two in Spain (25, 32), two in Germany (27, 36), two in China (9, 34), one in Austria (33), one in France (37), one in Italy (39), and one in America (41).

To assess depression, seven studies used the Beck Depression Inventory (BDI); three articles used the Zung Self-Rating Depression Scale (SDS); two studies used the Hamilton Depression Rating Scale (HAMD), two studies used the Hospital Anxiety And Depression Scale (HADS); and the remaining eight studies used screening tools including Goldberg scale, The Symptom Checklist-90-Revised (SCL-90-R), Center for Epidemiologic Studies for Depression (CES-D), QD2A, EuroQol five-dimensional questionnaire (EQ-5D), Patient Health Questionnaire-9 m (PHQ-9), Temperament Evaluation of Memphis, Pisa, Paris, and San Diego-Auto questionnaire (TEMPS-A) and Geriatric Depression Scale (GDS).

For the assessment of anxiety, six studies used the Beck Anxiety Inventory (BAI); three articles used the Zung Self-Rating Anxiety Scale (SAS); three used the Hamilton Anxiety Rating Scale (HAMA); two used the Hospital Anxiety and Depression Scale (HADS); two used Goldberg scale; and the remaining four studies used screening tools including SCL-90-R, State–Trait Anxiety Inventory (STAI), EQ-5D, and TEMPS-A.

### 3.3 Association of RLS with depression

**Figure 2:**
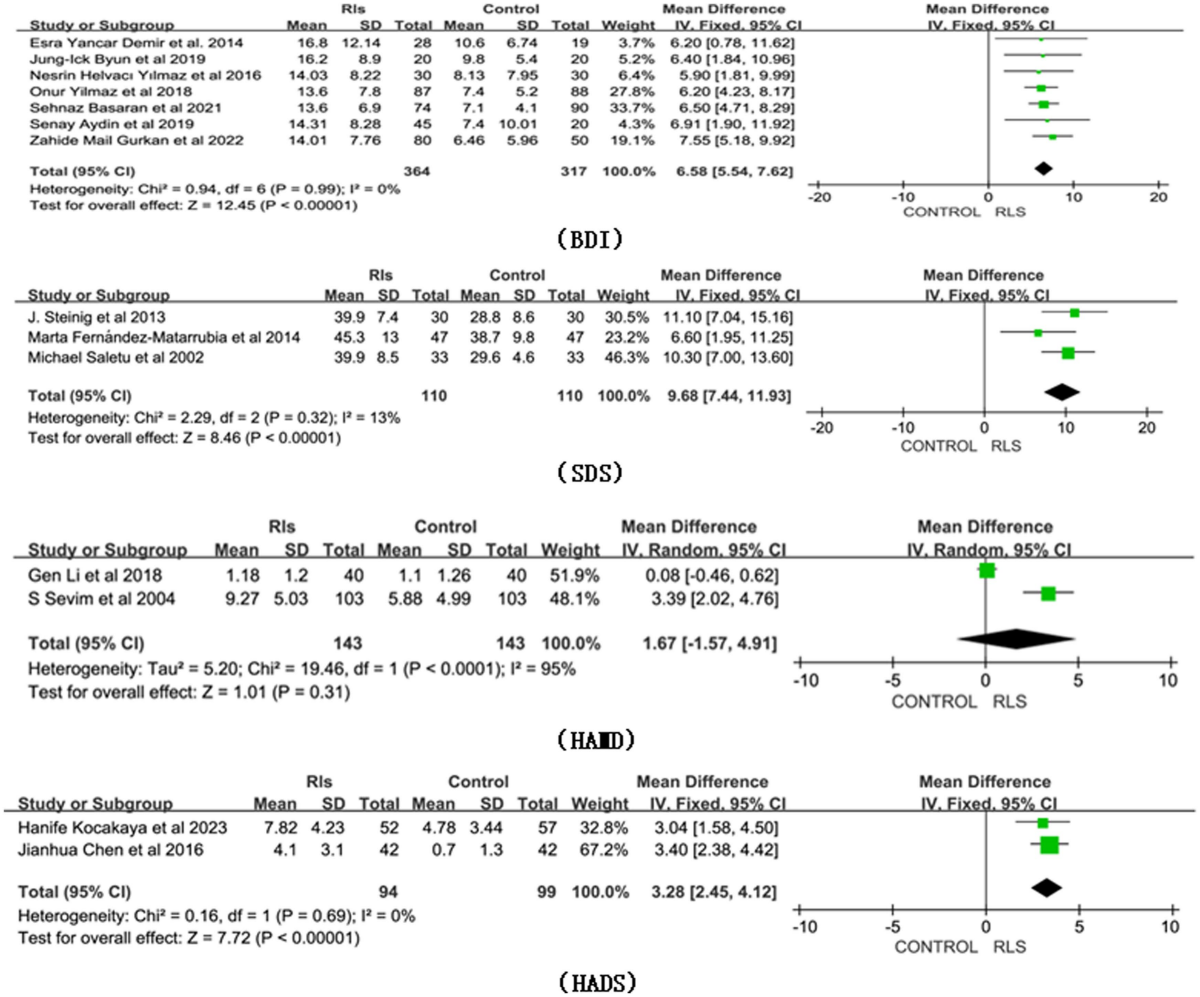
Forest plot of RLS and risk of depression symptoms. CI, Confidence interval; RLS, Restless Legs Syndrome.

As shown in Figure 2, a meta-analysis of cross-sectional, case–control, and cohort studies revealed a significant association of RLS with depression. Compared with healthy individuals (*n* = 317), individuals with RLS (*n* = 364) had higher scores on the BDI, based on the fixed model (MD 6.58, 95% CI 5.54, 7.62, Z = 12.45, *p* < 0.01, Heterogeneity: I^2^ = 0%; *p* = 0.99), which suggests they have more severe depression. In addition, SDS and HADS were also significantly higher in individuals with RLS compared to healthy controls (for SDS, MD 9.68, 95% CI 7.44, 11.93, Z = 8.46*, p <* 0.01; Heterogeneity: I^2^ = 13%; *p* = 0.32; and for HADS, MD 3.28, 95% CI 2.45, 4.12, Z = 7.72, *p* < 0.01, Heterogeneity: I^2^ = 0%, *p* = 0.69). However, based on the random model, HAMD did not show a significant difference in depression between individuals with RLS those without RLS (MD 1.67, 95% CI −1.57, 4.91, Z = 1.01, *p* = 0.31, Heterogeneity: I^2^ = 95%, *p* < 0.01).

### 3.4 Association of RLS with anxiety

**Figure 3:**
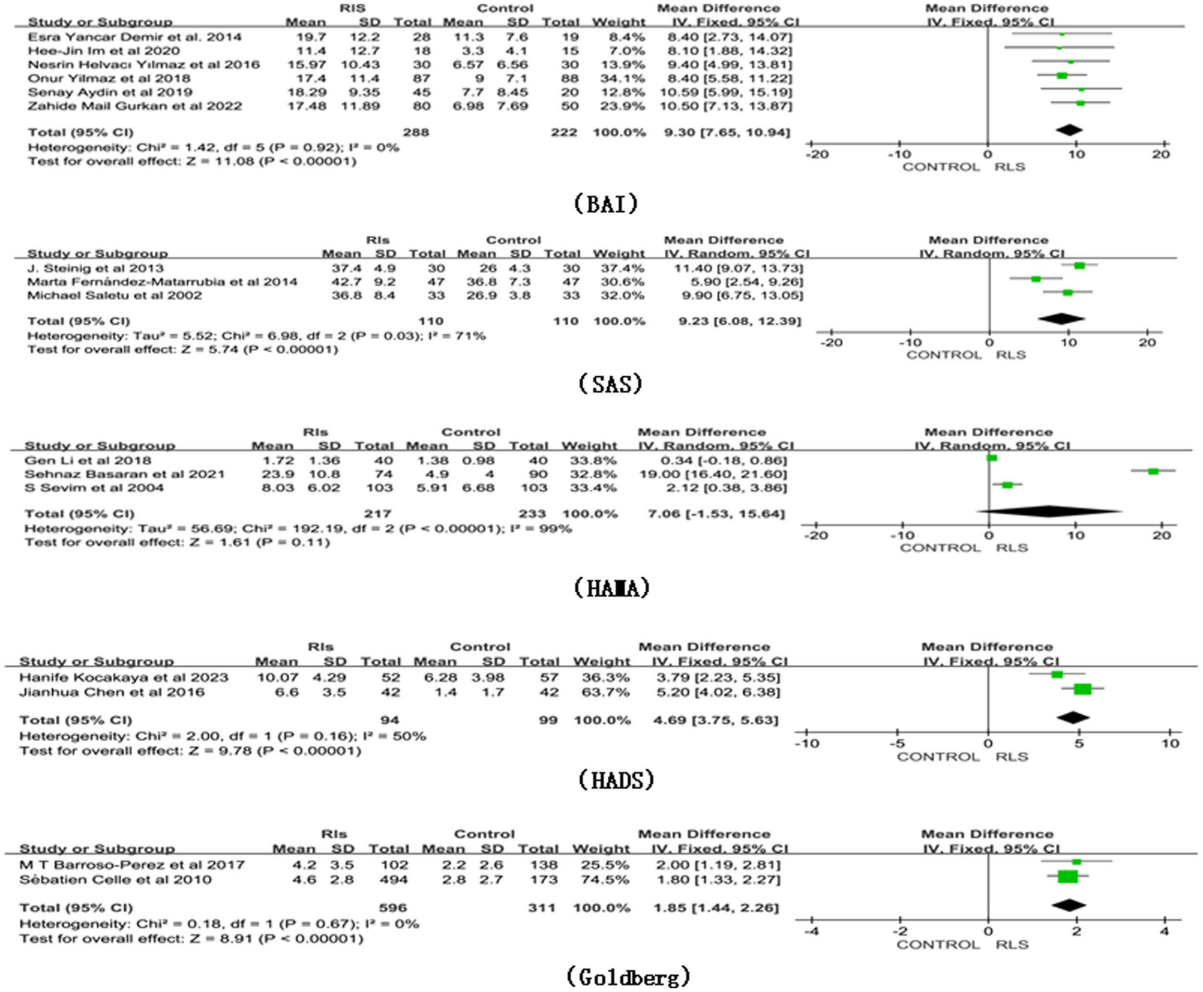
Forest plot of RLS and risk of anxiety symptoms. CI, Confidence interval; RLS, Restless Legs Syndrome.

Compared with healthy controls (*n* = 222), individuals with RLS (*n* = 288) had higher scores on the BAI, based on the fixed model (Figure 3: MD 9.30, 95% CI 7.65 10.94, Z = 11.08, *p* < 0.01, Heterogeneity: I^2^ = 0%, *p* = 0.92). Moreover, SAS, HADS, and Goldberg scores were all significantly higher in individuals with RLS compared to healthy controls (respectively, MD 9.23, 95% CI 6.08, 12.39, Z = 5.74, *p* < 0.01, Heterogeneity: I^2^ = 71%, *p* = 0.03, MD 4.69; 95% CI 3.75, 5.63, Z = 9.78 *p* < 0.01, Heterogeneity: I^2^ = 50%; *p* = 0.16, and MD 1.85; 95% CI 1.44, 2.26, Z = 8.91 *p* < 0.01, Heterogeneity: I^2^ = 0%, *p* = 0.67). However, based on the random model, HAMA did not show a significant difference in anxiety scores for RLS (MD 7.06, 95% CI -1.53, 15.64, Z = 1.61, *p* = 0.11, Heterogeneity: I^2^ = 99%, *p* < 0.01).

### 3.5 Heterogeneity across studies, publication bias, subgroup analysis, and sensitivity analysis

**Figure 4:**
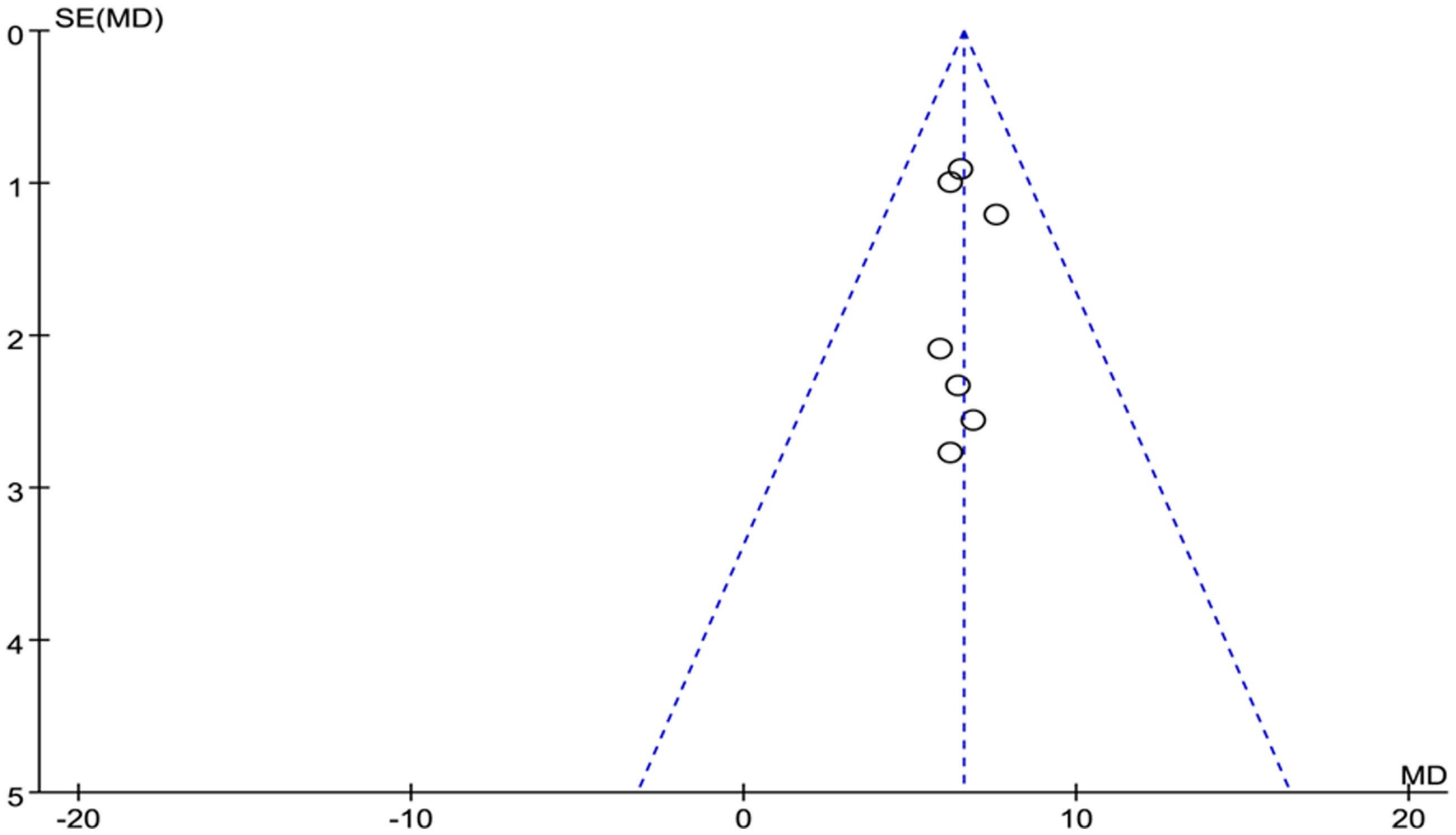
The publication bias of funnel panel on depression symptoms was measured by BDI.

**Figure 5:**
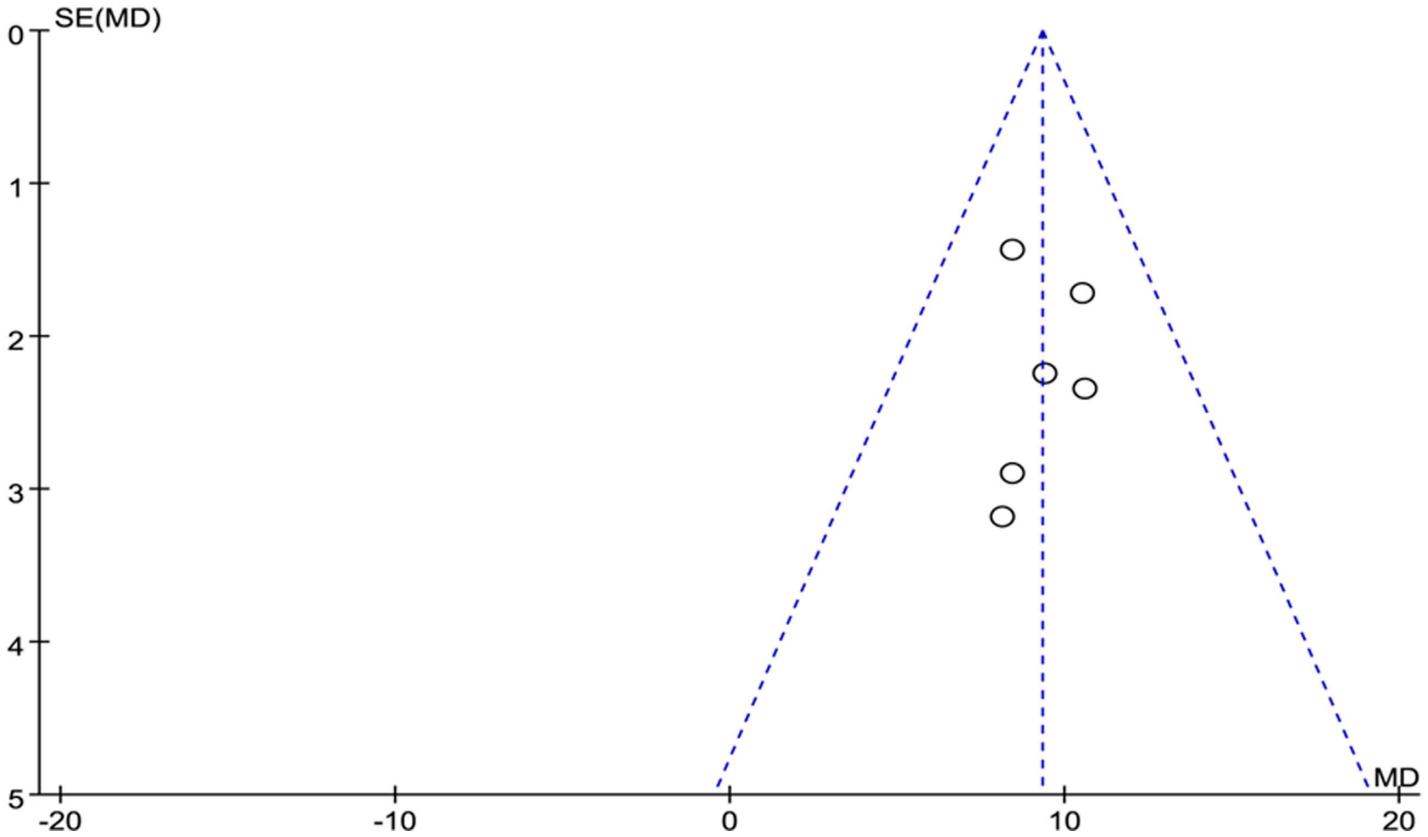
Funnel panel for publication bias of depression symptoms was measured by BAI.

**Figure 6:**
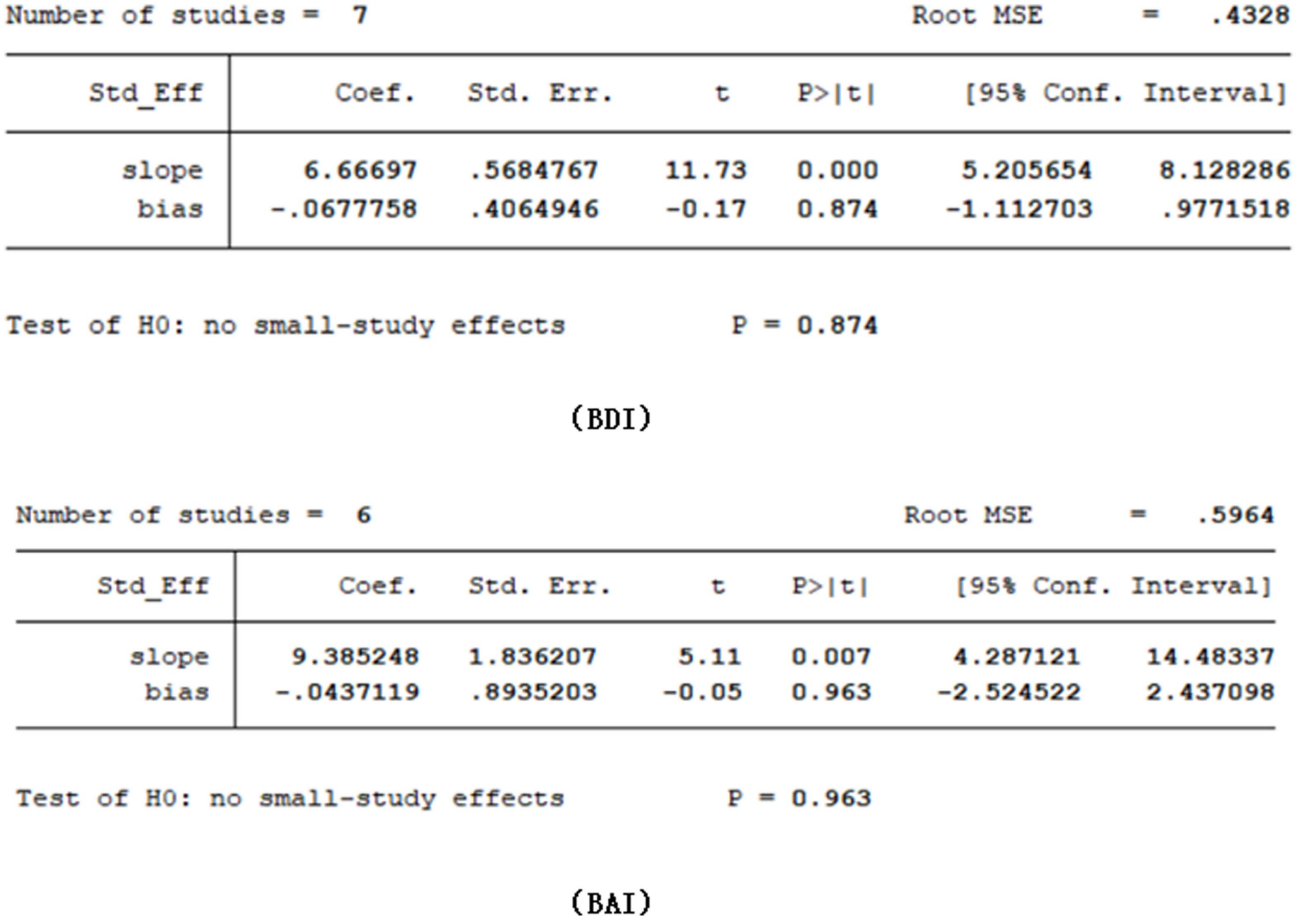
Egger test results.

**Figure 7:**
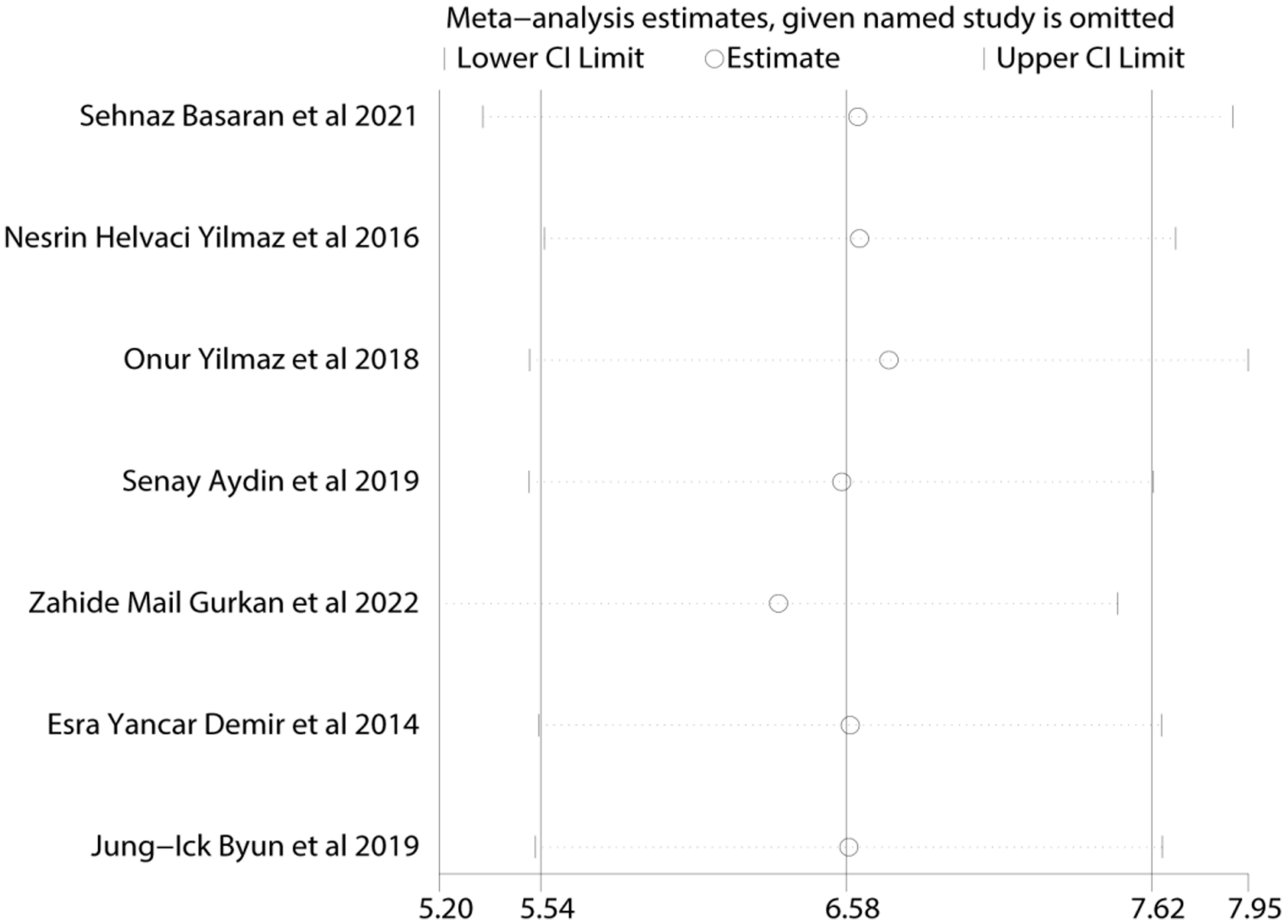
Sensitivity analysis of depression symptoms was measured by BDI.

**Figure 8:**
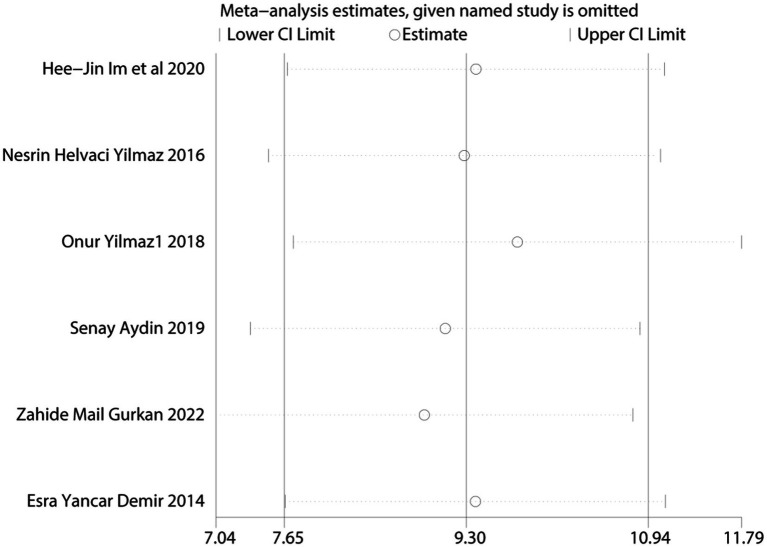
Sensitivity analysis of anxiety symptoms was measured by BAI.

Given the limited inclusion of literature, we focused our primary analysis on the two scales that featured the most comprehensive literature, namely BDI and BAI. Because no statistically significant heterogeneity (defined as a *p* value of X^2^ < 0.1 or I^2^ > 50%) across studies was detected, we opted for the fixed model for BDI and BAI. Visual inspection of the funnel plots indicated a low publication bias (Figures 4, 5). The Egger’s test results for BDI (*p* = 0.874) and BAI (*p* = 0.963) were non-significant, suggesting no publication bias (Figure 6) (42). Sensitivity analyses were performed by excluding one study each time and rerunning the analysis to verify the robustness of the overall results. A sensitivity analysis confirmed the robustness of our significant findings of the result from BDI and BAI (Figures 7, 8). Subgroup analysis of RLS and healthy controls was used for HAMA was shown in Table 3.

**Table 3:** Subgroup analysis of RLS and healthy controls was used for HAMA.

Subgroup factors	No. of studies	No. of patients	Effect model	95%CI	P	Heterogeneity
Total	3	271	Random	7.06[−1.53, 15.64]	0.11	I^2^ = 99% *p* < 0.0.1
**Areas**						
Turkey	2	177	Random	10.53[−6.01, 27.07]	0.21	I^2^ = 99% *p* < 0.0.1
China	1	40	**-**	0.34[−0.18,0.86]	0.20	-
**Age**						
≤50	2	177	Random	10.53[−6.01,27.07]	0.21	I^2^ = 99% *p* < 0.0.1
>50	1	40	-	0.34[−0.18,0.86]	0.20	-
**Sample size**						
≤100	2	114	Random	9.62[−8.66,27.91]	0.30	I^2^ = 99% *p* < 0.0.1
>100	1	103	-	2.12[0.38,3.86]	0.02	-
**Percentage of female**						
≥70%	2	143	Random	1.03[−0.67,2.73]	0.24	I^2^ = 73% *p* = 0.05
<70%	1	74	-	19.00[16.40,21.60]	*P* < 0.01	-
**RLS**						
Primary	2	114	Random	9.62[−8.66,27.91]	0.30	I^2^ = 99% *p* < 0.0.1
primary + secondary	1	103	-	2.12[0.38,3.86]	0.02	-

## 4 Discussion

This study summarized and compared the current evidence of depression and anxiety symptoms between two group: a group with RLS and a group without RLS. A total of 21 studies were systematically reviewed and subjected to meta-analysis. Despite variations in sample sizes and the diagnostic scales used for depression, anxiety, or both, the selected studies demonstrated high overall methodological quality. However, certain results exhibited significant heterogeneity. We found that the levels of depressive or anxious symptoms were significantly higher in individuals with RLS than in those without RLS. To the best of our knowledge, this is the first meta-analysis to quantitatively compare levels of anxiety and depression between adults with RLS and healthy controls.

The association between RLS and anxiety or depression levels in individuals with other medical conditions has previously shown mixed results. Some studies found that RLS in conjunction with other medical conditions was associated with higher anxiety or depression levels. Recent studies based on large cross-sectional survey data suggested that RLS in individuals with multiple sclerosis (MS) may contribute to heightened anxiety and depression symptoms. The effects of RLS on patients with MS included negative effects on functional ability, anxiety levels, sleep quality, and health-related quality of life (43, 44). Rana et al. showed that individuals with Parkinson’s disease combined with RLS had the highest incidence of anxiety and depression (45). Lee et al. found that untreated patients with idiopathic RLS and periodic limb movement during sleep had high levels of anxiety and depression (46). Recent studies in patients with cancer have suggested a significant increase in anxiety and depression levels, as evidenced by higher total HADS scores in those with RLS (47, 48). However, RLS in other diseases does not consistently correlate with anxiety and depression. There was no significant difference in anxiety and depression levels observed in adults with attention-deficit hyperactivity disorder or inflammatory bowel disease who also had RLS (49, 50). The use of varying scales of measures to compare the anxiety and depression levels of individuals with RLS with healthy controls may lay the groundwork for understanding complex associations. Building on this knowledge gap, we conducted a meta-analysis focusing on RLS-related anxiety and depression. By comparing the results from various depression and anxiety scales, our analysis demonstrated that the symptoms of depression or anxiety were more pronounced in individuals with RLS than in individuals without RLS.

In addition, data related to the interaction between RLS severity and anxiety and depression in patients with RLS are conflicting. One study from Korea evaluated anxiety and insomnia as factors that affected RLS severity (51). Other studies have shown no relationship between RLS severity and differences in depression and anxiety. Depressive symptoms in patients with RLS seem to be related to sleep impairment. Subjects with chronic insomnia were at a high risk of developing major depressive disorder (52–54). Perhaps more severe RLS symptoms associated with sleep disturbance would have had an effect on cognitive performance if left untreated (39, 55). A future meta-analysis could continue to examine the contribution of RLS severity to the increased risk of anxiety and depressive symptoms.

In the meta-analysis, the HAMD and HAMA did not show statistically significant differences between the individuals with RLS and the controls. First, it is important to note that the HAMD was only used in the studies reported in two articles; therefore, the number of articles was too small to confer certainty to the results. Second, significant heterogeneity was observed in the HAMA scores. Heterogeneity remained after subgroup analysis, and differences in age range, study design (including psychological assessment), scoring criteria, and sample size may have contributed to this heterogeneity.

Our findings highlight the positive associations between RLS and depression and anxiety. The precise mechanisms underlying these associations have not been elucidated. Nonetheless, certain evidence might help explain this phenomenon. The patients with RLS experienced night-time sensations characterized by an irresistible urge to move their legs. These sensations intensified during periods of rest, particularly at night, leading the patients to engage in movements or massage their limbs for relief. Prolonged occurrences of these symptoms significantly diminished the sleep quality of individuals with RLS, potentially triggering the onset of depression and anxiety (56). Dopamine is a neurotransmitter involved in muscle control and behavior and has a crucial role in the functioning of the central nervous system. When dopamine-pathway dysfunction occurs, involuntary movement can occur, which could lead to RLS (57–59). Our review showed that dopamine levels followed a circadian pattern, with the highest levels observed at 8 a.m. In the evening, between 20:00 and 22:00, dopamine levels dropped to approximately 60% of their peak levels. The lowest point occurred at 3 am (60). Therefore, the patients had mild RLS symptoms during the day and severe symptoms at night. Decreased levels of monoamine neurotransmitters, such as dopamine, could lead to anxiety and depression (61, 62). At night, dopamine levels were low, which may have caused anxiety and depression. Meanwhile, the patients with RLS had symptoms of severe physical discomfort, and their uncomfortable symptoms might aggravate anxiety and depression. RLS may have a substantial psychological effect on the affected individuals. Our study revealed a need in the clinical setting for physicians to evaluate psychological symptoms when diagnosing RLS and consider incorporating psychological counseling for patients as part of the treatment.

The current study had some limitations. First, not all depression and anxiety scales were analyzed, with some scales having too few associated literature articles for a comprehensive analysis, such as the STAI, PHQ-9, GDS, and SCL-90-R. Second, although we conducted subgroup analyses, the identified heterogeneity remained high, which made it challenging to pinpoint the exact source. Future studies with larger sample sizes are warranted to clearly identify the sources of heterogeneity.

## 5 Conclusion

This systematic review and meta-analysis of cross-sectional, case–control, and cohort studies revealed a higher risk of depression and anxiety among individuals with RLS than among their healthy counterparts. Efforts should be made by organizations to increase awareness of the psychological health of individuals affected by RLS.

## Data availability statement

The original contributions presented in the study are included in the article/supplementary material, further inquiries can be directed to the corresponding author.

## Author contributions

TA: Formal analysis, Methodology, Software, Writing – original draft. HS: Formal analysis, Methodology, Software, Writing – review & editing. LY: Methodology, Software, Writing – review & editing. XW: Formal analysis, Methodology, Writing – review & editing. BL: Funding acquisition, Methodology, Writing – review & editing.
